# Ecological Dynamics of Two Distinct Viruses Infecting Marine Eukaryotic Decomposer Thraustochytrids (Labyrinthulomycetes, Stramenopiles)

**DOI:** 10.1371/journal.pone.0133395

**Published:** 2015-07-23

**Authors:** Yoshitake Takao, Yuji Tomaru, Keizo Nagasaki, Daiske Honda

**Affiliations:** 1 Department of Marine Bioscience, Fukui Prefectural University, 1–1 Gakuencho, Obama, Fukui, 917–0003, Japan; 2 National Research Institute of Fisheries and Environment of Inland Sea, Fisheries Research Agency, 2-17-5 Maruishi, Hatsukaichi, Hiroshima, 739–0452, Japan; 3 Department of Biology, Faculty of Science and Engineering, Konan University, 8-9-1 Okamoto, Higashinada, Kobe, 658–8501, Japan; CSIR- National Institute of Oceanography, INDIA

## Abstract

Thraustochytrids are cosmopolitan osmotrophic or heterotrophic microorganisms that are considered as important decomposers in coastal ecosystems. However, because of a lack of estimation method for each genus or systematic group of them, relatively little is known about their ecology *in situ*. Previously, we reported two distinct types of virus infecting thraustochytrids (AuRNAV: reported as SssRNAV, and SmDNAV) suggesting they have wide distributions in the host-virus systems of coastal environments. Here we conducted a field survey from 2004 through 2005 to show the fluctuation pattern of thraustochytrids and their viruses in Hiroshima Bay, Japan. During the field survey, we monitored the dynamics of the two types of thraustochytrid-infecting virus: small viruses causing lysis of *Aurantiochytrium* sp. NIBH N1-27 (identified as AuRNAV) and the large viruses of *Sicyoidochytrium minutum* NBRC 102975 (similar to SmDNAV in physiology and morphology). Fluctuation patterns of the two distinct types of virus were different from each other. This may reflect the difference in the preference of organic substrates; i.e., it may be likely the host of AuRNAV (*Aurantiochytrium* sp.) increases utilizing algal dead bodies or feeble cells as the virus shows a large increase in abundance following raphidophyte blooms; whereas, the trophic nutrient supply for *S*. *minutum* may primarily depend on other constantly-supplied organic compounds because it did not show any significant change in abundance throughout the survey. Further study concerning the population composition of thraustochytrids and their viruses may demonstrate the microbial ecology (especially concerning the detrital food web) of marine environments.

## Introduction

Thraustochytrids are non-photosynthetic marine/estuarine stramenopile protists that are frequently observed and/or isolated from marine and estuarine waters, sediments, algal and plant materials both as saprotrophs and parasites [1]. Their bio-volume in coastal waters is estimated at 43% of that of the bacterioplankton [2]. The ubiquitousness and ability to use a wide variety of organic substrates (including bacterivory) argue for their ecological importance as decomposers [3, 4]. In addition, due to their high production of PUFAs (polyunsaturated fatty acids) such as docosahexaenoic acid and docosapentaenoic acid [5], they are considered very important as food resources for higher organisms in marine systems [6–8]. Because of these distinctive features of the thraustochytrids, their ecological significance in the coastal ecosystems has been studied [9, 10]. Kimura et al. [11] biogeographically demonstrated the abundance of thraustochytrids was closely related with the density of POM (particulate organic matter). And Bongiorni and Dini [12] show the abundance and composition of thraustochytrids change with habitats and seasons in Mediterranean coastal areas. However, the effective techniques that can separately estimate the abundance of each genus or systematic groups of thraustochytrids, still have not been established. In spite of their ecological significance, therefore, relatively little is known about their ecological influence and impact *in situ*.

On the other hand, viruses are highly abundant in marine environments and are recognized as important pathogens in controlling bacterial and algal biomass [13, 14], nutrient cycling [15], and in maintaining the bio-diversity of bacteria and microalgae [14, 16]. To date, more than thirty algal viruses have been isolated and characterized to different levels of resolution; and particularly, the relationships between algal blooms and viruses have been intensively investigated [17]. The viral infection is considered to affect the dynamics of algal blooms both quantitatively (biomass) and qualitatively (clonal composition). *Heterosigma akashiwo*-HaV(*Heterosigma akashiwo* virus) and *Heterocapsa circularisquama*-HcRNAV (*Heterocapsa circularisquama* RNA virus) are well known host-virus systems [18, 19]. In both cases, the host and their virus dynamics were tightly linked each other [20, 21]. Considering the fact that viruses can’t reproduce without their specific host, fluctuations in abundance of certain virus may reflect the host dynamics. Therefore, studies on viruses that infect thraustochytrids lead up to novel information about their host.

Previously, we reported two distinct viruses infecting thraustochytrids: AuRNAV (*Aurantiochytrium* RNA virus: reported as SssRNAV) and SmDNAV (*Sicyoidochytrium minutum* DNA virus) [22, 23]. AuRNAV is a single-strand RNA virus infecting *Aurantiochytrium* sp. (formerly Schizochytrium sp., see Yokoyama and Honda, [24]); and SmDNAV is a double-strand DNA virus infecting *S*. *minutum*. The two hosts are taxonomically distant within the family Thraustochytriaceae. Here we describe the seasonal change in abundance of viruses infecting the thraustochytrids in Hiroshima Bay, Japan and discuss the ecology of thraustochytrids from the viewpoint of the host-virus relationships.

## Materials and Methods

### Sample collection

**Fig 1:**
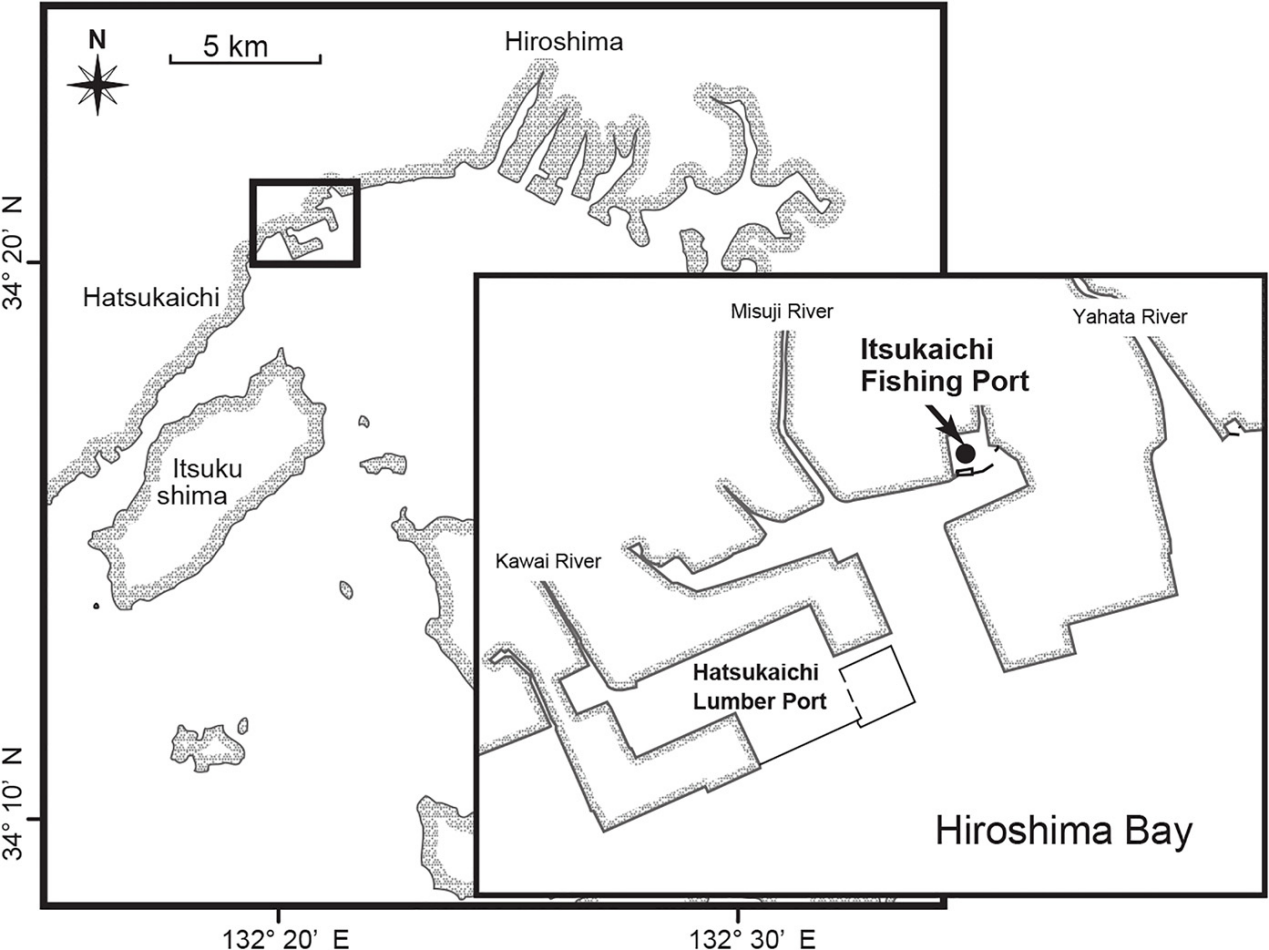
Site map of the sampling location in Hiroshima Bay, Japan. This map was originally drawn by using Adobe Illustrator (Adobe Systems Software Ireland Ltd.) based on the geological information.

Water samples from the surface layer (0 m) and 0.2 m above the sediment-water interface (B-0.2 m) were collected in 2004 (May—Dec) and 2005 (Apr—Aug) at a semi-enclosed basin (Itsukaichi Fishing Port; 34°21.400N, 132°21.864E, mean water depth = 5 m) located in Hiroshima Bay, Japan (Fig 1). No specific permits were required for the described field studies, as the location is not privately-owned or protected in any way, and the field studies did not involve endangered or protected species.

During the survey, the water temperature and salinity (psu) of the water column ranged at 16.4–28.1°C and 16.6–30.3, respectively.

### Abundance of phytoplankton and thraustochytrids

Phytoplankton abundance was enumerated by direct count using light microscopy. During the 2005 survey, the abundance of thraustochytrids was estimated using a modified MPN (most probable number) technique with pine pollen [25].

### Virus titration

Virus titration of seawater samples (0 m and B-0.2 m) and sediment samples was conducted using the extinction dilution method [26]. Six thraustochytrid strains were grown at 20°C in 10×medium-H [27] and were used as titration host strains for thraustochytrids-infecting viruses in the water samples (Table 1). The cell culture plates were incubated at 20°C. The occurrence of cell lysis was monitored every other day for 14 days using optical microscopy; and the most probable number (MPN) of viruses lytic to each host strain was calculated using the computer program of Nishihara et al. [28]. Thus, viral abundance was estimated as the MPN of infectious units that are lytic to each host strain. Cell lysates in the most diluted wells were filtered through a 0.2-μm filter and then forwarded to the following isolation procedure.

**Table 1:** Host strains used to detect infecting viruses.

Taxon	Strains*	Isolation locality
Thraustochytriaceae	*Aurantiochytrium* sp. NIBH N1-27	Nakaminato Harbor, Ibaragi, Japan
	*Aurantiochytrium limacinum* NIBH SR-21	Colonia, Yap Island, Micronesia
	*Schizochytrium* sp. SEK 0213	Iriomote Island, Okinawa, Japan
	*Sicyoidochytrium minutum* NBRC 102975	Iriomote Island, Okinawa, Japan
	*Thraustochytrium aureum* ATCC 34304	Woods Hole, Massachusetts, USA
	*Ulkenia amoeboidea* SEK 0214	Hiroshima Bay, Hiroshima, Japan

*ATCC: American Type Culture Collection (USA), NBRC: National Institute of Technology and Evaluation (NITE) Biological Research Center (Japan), NIBH: National Institute of Bioscience and Human-Technology (Japan), SEK: Laboratory of Systematics and Evolution, Konan University (Japan).

### Isolation of viruses

One clonal virus was isolated from each MPN assay. Clonal pathogens were obtained using two cycles of the extinction dilution procedure [29] with the same thraustochytrid strain that was initially used for titration. The resultant lysate at the final end-point dilution series from the second extinction dilution procedure was regarded as a clonal virus suspension in which the probability of two or more differing viruses occurring (i.e., failure in cloning) was estimated at p ≤ 0.0106. The clonal viral suspensions were made free from bacterial contamination using filtering through a 0.2-μm filter. The virus clones successfully isolated and free from bacterial contamination were stored with 10% glycerol in 10×medium-H at -80°C.

### TEM observation

TEM observation of the isolated viral clones was performed to show their morphology. Negatively stained viruses were prepared according to the method of Takao et al. [22]; and observed using TEM at an acceleration voltage of 80 kV using a JEOL JEM-1010 transmission electron microscope. Particle sizes were estimated from negatively stained images.

### Northern dot-blot analysis

Northern dot-blot analysis was performed to identify whether the viral isolate causing lysis of *Aurantiochytrium* sp. NIBH N1-27 was AuRNAV (or a closely-ralated virus). An AuRNAV-specific probe was synthesized using the AlkPhos Direct Labelling and Detection System with CDP-Star (GE Healthcare) and the cDNA of AuRNAV including the non-coding and ORF2 region (nt 5231–5834; GenBank accession number AB193726) as the template [30]. Each virus clone was inoculated into its suitable host strain (*Aurantiochytrium* sp. NIBH N1-27) and incubated for 48 h. This is long enough for the tested-viruses to complete one replication cycle as previously evaluated [22]. Two-μl of each viral suspension was dotted onto a positively-charged nylon membrane, Hybond-N^+^ (Amersham Biosciences); then, hybridization was performed according to the manufacturer's protocol using the above mentioned AuRNAV-specific probe; and detected with chemical illumination using a LAS-3000 (Fuji Photo Film Co., Ltd.)

## Results

### Isolation and identification of viruses

We succeeded in isolating 118 viral isolates infecting thraustochytrids during the field surveys conducted in 2004 and 2005. Among the clones, 52 and 66 were infectious to *Aurantiochytrium* sp. NIBH N1-27 and *S*. *minutum* NBRC 102975, respectively. TEM observation revealed the former were small icosahedral viruses similar to AuRNAV (ca. 25 nm in diameter, angular in shape and lacking a tail) and the latter were large ellipse viruses resembling SmDNAV in morphology (ca. 150 nm in diameter and lacking a tail) (S1 Fig). Northern dot-blot analysises revealed all of the 52 AuRNAV-like viral isolates showed positive reaction to the specific molecular probes, respectively (S2 Fig). Integrating the results of TEM observation with northern dot-blot analysis, all of the isolated viral clones that caused lysis of *Aurantiochytrium* sp. NIBH N1-27 were identified as AuRNAV [22]; whereas, those lytic to *S*. *minutum* NBRC 102975 were considered to be (or closely related to) SmDNAV [23].

### Field data collected in 2004

**Fig 2:**
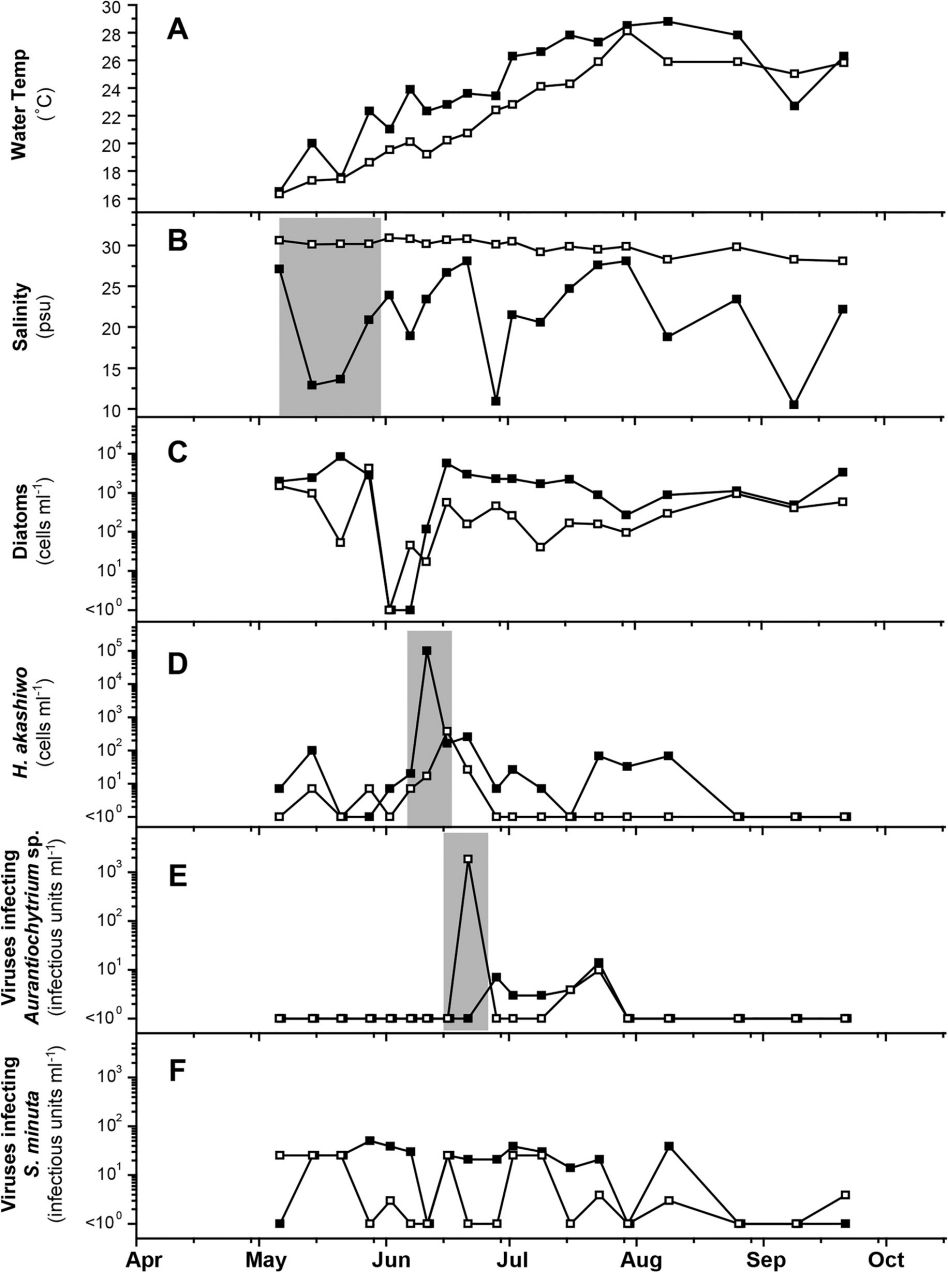
Temporal changes in water temperature (A), salinity (B), and abundance of diatoms (C), *Heterosigma akashiwo* (D), viruses infecting *Aurantiochytrium* sp. NIBH N1-27 (E), and viruses infecting *Sicyoidochytrium minutum* NBRC 102975 (F) during the field survey at Hiroshima Bay in 2004. (■) and (□) indicate data at the surface and the B-0.2 m (0.2 m above the sediment-water interface) layer, respectively. Remarkable peaks and valleys were highlighted by shadowing.

The temporal changes in the field data collected during 2004 are shown in Fig 2. Significant decreases in practical salinity units (psu) were observed three times; in May, Jun, and Aug (Fig 2). The diatoms in the genera *Chaetoceros* and *Skeletonema* dominated in the water column almost throughout the survey period except during a *Heterosigma akashiwo* bloom where the highest cell density was 9.7 × 10^4^ cells ml^-1^ on Jun 11 (Fig 2).

Six phylogenetically distant thraustochytrid clones were used to isolate and enumerate their viruses throughout the survey; and consequently, only two types of virus respectively infecting *Aurantiochytrium* sp. NIBH N1-27 (here regarded as AuRNAV) and *Sicyoidochytrium minutum* NBRC 102975 (SmDNAV-like viruses) were detected and successfully isolated (Table 1 and Fig 2). AuRNAV showed a drastic increase in abundance (1.8 × 10^3^ infectious units ml^-1^) at ten days after the *H*. *akashiwo* bloom termination. Then, they rapidly decreased to below the detection limit at 3.0 infectious units ml-^1^ after 23 Jul (Fig 2). Whereas, the viruses infecting SmDNAV-like viruses did not show any changes in abundance during the survey period which fluctuated at <2.5 × 10^1^ infectious units ml-1 (Fig 2).

Considering the fact that viruses can’t reproduce without their specific host, fluctuations in abundance of certain virus may reflect the host dynamics. In addition, our preliminary inoculation experiments showed that *Aurantiochytrium* sp. NIBH N1-27 attached to *H*. *akashiwo* cells and propagated more efficiently than other thraustochytrid strains tested (data not shown). Therefore, we thought that the spike peak of thraustochytrids existed after the *H*. *akashiwo* bloom but before the peak of AuRNAV. To confirm the hypothesis, we also estimated the abundance of thraustochytrids during the field survey in 2005.

### Field data collected in 2005

**Fig 3:**
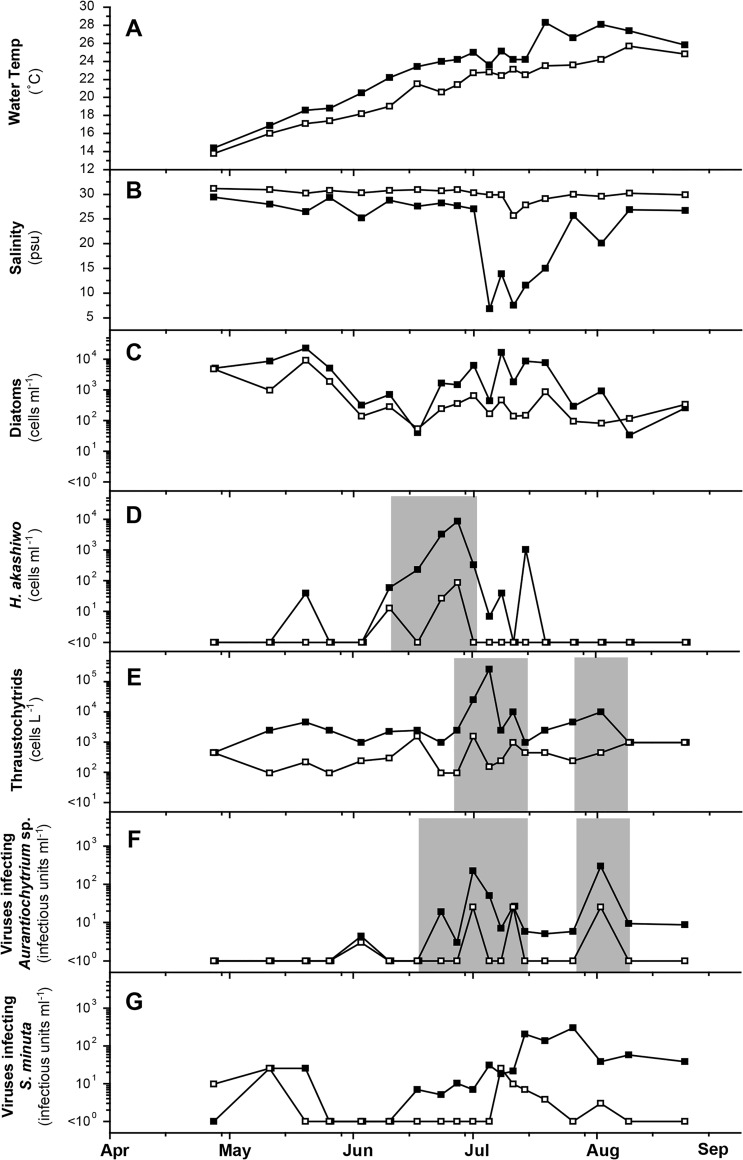
Temporal changes in water temperature (A), salinity (B), and abundance of diatoms (C), *Heterosigma akashiwo* (D), thraustochytrids (E), viruses infecting *Aurantiochytrium* sp. NIBH N1-27 (F), and viruses infecting *Sicyoidochytrium minutum* NBRC 102975 (G) during the field survey at Hiroshima Bay in 2005. (■) and (□) indicate data at the surface and the B-0.2 m layer, respectively. Remarkable peaks and valleys were highlighted by shadowing.

Temporal changes in field data collected in 2005 are shown in Fig 3. During 2005, there was little precipitation during the rainy season (May to Jun) and a decrease in salinity was detected only in Jul (Fig 3). Similar tendencies in algal dominance as in 2004 were also observed in 2005; i.e., diatoms such as genera Chaetoceros and Skeletonema were usually dominant in the water column except during a *H*. *akashiwo* bloom and short period dominance of *Thalassiosira* spp. (14–19 Jul) (Fig 3). *H*. *akashiwo* bloom (smaller but longer than 2004) was detected with a maximum cell density of 8.8 × 10^3^ cells ml^-1^ during Jun 23 to 27 (Fig 3).

In 2005, only the two similar types of virus were detected as in the case of 2004 (Fig 3); however, the fluctuation pattern differed from the preceding year. The abundance of AuRNAV peaked (Jul 1; 2.2 × 10^2^ infectious units ml^-1^) at the end of the *H*. *akashiwo* bloom, and one month later (2 Aug; 3.0 × 10^2^ infectious units ml^-1^) (Fig 3). SmDNAV-like viruses fluctuated at less than 100 infectious units ml-1 from 26 May through 10 Jun, where the abundance was less than the detection limit; and then, they moderately increased and reached a maximum (3.0 × 10^2^ infectious units ml^-1^) on 26 Jul (Fig 3).

Changes in abundance of thraustochytrids were estimated in 2005 (Fig 3). The abundance ranged between 9.1 × 10^1^ to 2.3 × 10^5^ cells L^-1^. An increase accompanied with an increase of AuRNAV was observed at the end of the *H*. *akashiwo* bloom (from 23 Jun through 5 Jul) and was followed by a drastic decrease; then, they showed a moderate increase (Fig 3).

## Discussion

In the present field survey, we detected two distinct types of thraustochytrid virus; they were AuRNAV and SmDNAV-like. Considering both virus types were isolated from a variety of coastal environments in Japan, at least two distinct thraustochytrid-virus combinations may be widely distributed and functioning in universal eukaryotic decomposing systems. However, no viral agents causing lysis of the other four tested thraustochytrid clones were detected throughout the present survey. Of course, this does not deny the possibility of the existence of other thraustochytrid-infecting viruses. In this study, we used only six host strains to examine virus abundance; as a result, only the viruses which caused lysis of tested hosts were isolated. It may be possible to isolate a wider variety of viruses by using more thraustochytrid strains as hosts. As well, it should be noted that host-virus combinations not accompanied with drastic cell lysis may have been overlooked in this study.

The fluctuation patterns in abundance of the two virus types were different from each other. AuRNAV showed a remarkable increase in abundance following the *H*. *akashiwo* bloom in 2004 (Fig 2). Since AuRNAV does not infect *H*. *akashiwo* [22], the increase is considered reflecting the drastic increase and viral lysis of *Aurantiochytrium* sp. NIBH N1-27-type thraustochytrids, which occurred following the *H*. *akashiwo* bloom. Actually, we succeeded in detecting an increase in thraustochytrid abundance after the peak of the *H*. *akashiwo* bloom also in 2005, which was accompanied with the temporal increase of AuRNAV (Fig 3). Whereas, no statistically significant relationship was found between the abundance of thraustochytrids and AuRNAV from the Pearson's correlation coefficient analysis (data not shown).

A possible explanation for the ecological events which was observed in 2004 is reasonable; i.e., *H*. *akashiwo* rapidly multiplied due to the enough amount of nutrient supply originated from land water (Fig 2), and it caused drastic increase and dominance of *Aurantiochytrium* sp. NIBH N1-27-type thraustochytrids (utilizing *H*. *akashiwo* cells). The spike peak of AuRNAV detected in 2004 was considered as the result of virus infection to the dominant thraustochytrids. On the other hand, in 2005, land water supply was less than 2004 before the *H*. *akashiwo* bloom. Then, the bloom scale was not as large as in 2004 (Fig 3). Although the similar events should have occurred also in 2005, each event may have occurred at lower level, thus, the event sequence was not so obvious in 2005 compared to 2004.

Another possibility is difference in the species composition of thraustochytrids. Untapped organic matter remained in water column and/or released organic matter derived from virally lysed NIBH N1-27-type thraustochytrid cells were considered to be substrates for multiplication of the other types of thraustochytrid. At the peak of thraustochytrids detected in 2005, *Aurantiochytrium* sp. NIBH N1-27-type may not have been as dominant as in 2004. Although we checked the total thuraustochytrids abundance to grasp the tendency of their dynamics, the resolution quality was too low to verify the hypothesis. Techniques for separately estimating the abundance of each genus or systematic groups of thraustochytrids is essential.

Peaks of AuRNAV and thraustochytrids were also detected from 19 Jul through 2 Aug 2005 (Fig 3) following the period when diatoms (genus *Thalassiosira*) dominated (14–19 Jul). In this case, it is considered that *Aurantiochytrium* sp. NIBH N1-27-type thraustochytrids increased by utilizing Thalassiosira sp. cells. Gaertner [31] reported that Schizochytrium sp. cells parasitized on Thalassiosira sp. cells. In addition, our preliminary experiments showed that *Aurantiochytrium* sp. NIBH N1-27 attached and propagated on the surface of diatom cells, as was observed in the case of *H*. *akashiwo* cells (data not shown). These results suggest that *Aurantiochytrium* sp. NIBH N1-27-type thraustochytrids may have the ability to effectively utilize the dead cells or feeble cells of phytoplankton (ex. *Thalassiosira* sp.).

On the other hand, SmDNAV-like viruses were detected more frequently than AuRNAV throughout the field surveys. In 2004, they fluctuated at a relatively low level; and did not show an increase either during or after the *H*. *akashiwo* bloom (Fig 2), suggesting that the dynamics of *S*. *minutum* may depend on constantly supplied organic matter (ex. DOM/POM from land). In 2005, the dynamics of SmDNAV-like viruses showed a moderate increase in abundance at the surface layer after a drastic inflow of huge amount of river water (Fig 3), but the abundance was not as high as AuRNAV. To this end, the fluctuation pattern of SmDNAV-like viruses was different from that of AuRNAV. The difference is most likely due to the distinctive nutrient acquisition strategies between *S*. *minutum* NBRC 102975-type and *Aurantiochytrium* sp. NIBH N1-27-type thraustochytrids.

To our knowledge, this is the first report describing the seasonal changes in the abundance of thraustochytrids and their viruses. Thraustochytrids is large taxa classified as a family, nevertheless only little is known about the differences and diversity of their ecological features. Our results suggested that the community of thraustochytrids is comprised of plural groups that have different ecological features and affected differently impacted by viruses. Further detailed study focused on their internal population composition is necessary to reveal their ecological impact *in situ* as a marine eukaryotic decomposer.

## Supporting Information

S1 Fig Transmission electron microscopy images of isolated viruses. AuRNAV (A), SmDNAV (B), small viruses infecting *Aurantiochytrium* sp. NIBH N1-27 (C and E), and large viruses infecting *Sicyoidochytrium minutum* NBRC 102975 (D and F).
(EPS)

S2 Fig Northern dot-blot analysis of viruses infecting *Aurantiochytrium* sp. NIBH N1-27. AuRNAV (A1), *Rhizosolenia setigera* RNA virus (A2), SmDNAV(A3), AuRNAV strains (MD002, MD003, KM006, KM007, IS004) isolated from other part of Japan coast area (A4–8), Viruses infecting *Aurantiochytrium* sp. NIBH N1-27 isolated in this study (A9—F10).
(EPS)
